# Numerical research on the lateral global buckling characteristics of a high temperature and pressure pipeline with two initial imperfections

**DOI:** 10.1371/journal.pone.0194426

**Published:** 2018-03-19

**Authors:** Wenbin Liu, Aimin Liu

**Affiliations:** 1 CCCC Tianjin Port Engineering Institute Co., Ltd., Tianjin, Tianjin, China; 2 CCCC First Harbor Engineering Co., Ltd., Tianjin, Tianjin, China; Seoul National University, REPUBLIC OF KOREA

## Abstract

With the exploitation of offshore oil and gas gradually moving to deep water, higher temperature differences and pressure differences are applied to the pipeline system, making the global buckling of the pipeline more serious. For unburied deep-water pipelines, the lateral buckling is the major buckling form. The initial imperfections widely exist in the pipeline system due to manufacture defects or the influence of uneven seabed, and the distribution and geometry features of initial imperfections are random. They can be divided into two kinds based on shape: single-arch imperfections and double-arch imperfections. This paper analyzed the global buckling process of a pipeline with 2 initial imperfections by using a numerical simulation method and revealed how the ratio of the initial imperfection’s space length to the imperfection’s wavelength and the combination of imperfections affects the buckling process. The results show that a pipeline with 2 initial imperfections may suffer the superposition of global buckling. The growth ratios of buckling displacement, axial force and bending moment in the superposition zone are several times larger than no buckling superposition pipeline. The ratio of the initial imperfection’s space length to the imperfection’s wavelength decides whether a pipeline suffers buckling superposition. The potential failure point of pipeline exhibiting buckling superposition is as same as the no buckling superposition pipeline, but the failure risk of pipeline exhibiting buckling superposition is much higher. The shape and direction of two nearby imperfections also affects the failure risk of pipeline exhibiting global buckling superposition. The failure risk of pipeline with two double-arch imperfections is higher than pipeline with two single-arch imperfections.

## Introduction

Pipeline global buckling is one of the major issues in pipeline design [1], especially for deep-water pipelines. The initial deflection, which is called the initial imperfection, inevitably exists in pipeline systems due to manufacture defects or the influence of uneven seabed [2], and the distribution of imperfections is random. The initial imperfections will trigger the buckling [3], and pipelines with multiple initial imperfections may experience the superposition of global buckling.

There are many studies on the buckling process of pipelines with one initial imperfection. Taylor and Gan [4] proposed the single-arch and double-arch imperfections considering the variation of soil resistance, and deduced the analytical solutions for lateral buckling. Taylor et al. [5] analyzed the vertical buckling of pipelines by using an analytical method and carrying out model tests, proposed three initial imperfection forms, and calculated the critical buckling force for pipelines with these imperfection forms. Croll [6] studied the vertical global buckling of pipelines with a simplified model considering the influence of initial imperfections and residual stress, and proposed formulas to calculate the critical buckling force of pipeline vertical buckling. Wantland el al. [7] analyzed the lateral stability of submerged pipelines in soft clay with data from laboratory and field investigations using model pipelines in cohesive soils. Preston et al. [8] studied the snake lay pipeline methodology and the performance of snake laid pipelines. Sriskandarajah et al. [9] defined realistic initial imperfections for buckling analysis and discussed how the parameters of initial imperfections affect the buckling. Villarraga et al. [10] established the non-linear numerical model to simulate the vertical buckling process of an imperfect pipeline with non-linear soil resistance. Bruton et al. [11] revealed that the axial force, geometry initial imperfection and soil resistance are the major impact factors on global buckling based on numerical simulation results. Sparks [12] payed attention to the influence of tension, pressure and weight on a pipeline’s deformations and stresses, and the concepts of effective tension and effective stress were redefined. Fyrileiv and Collberg [13] proposed the concept of effective axial force and discussed its use in offshore pipeline design in general and in DNV codes in particular. Bruton et al. [14–15] noted that the pipe-soil interaction has a significant influence on the pipeline design process and the uncertainty in pipe-soil resistance severely complicates pipeline design. Sun and Jukes [16] used “Simulator”, an ABAQUS based in-house Finite Element Analysis (FEA) engine, to establish a numerical simulation model to analyze the behavior of the pipeline from installation to operation. Chee and Walker [17] analyzed pipeline global buckling with 4 different numerical simulation methods, and results showed that with increasing imperfection amplitude, the maximum compressive strain decreases and the lateral displacement increases. There are two methods for initial imperfection introduction in numerical simulation. One way is to draw the initial imperfection in the model establishment of a pipeline; Konuk [18] and Newson [19] both used this method in simulation. Haq and Kenny [20] proposed parameter study results using calibrated numerical modeling procedures. The other way is to first calculate the most likely imperfection based on the Modal Analysis Method, and then introduce this imperfection in simulation by editing the keywords of models. Liu et al. [21] and Hong et al. [22–23] used this method. Li et al. [24] deduced analytical solution for solving global buckling of pipeline with distributed buoyancy sections. Zhang [25] analyzed pipeline buckling with finite element method software Patran and Abaqus and confirmed the reliability of FEA results by comparing with full-scale experiment. Sriskandarajah et al. [26] based on engineering observed data to predict pipeline lateral buckling deformation. Hakim et al. [27] carried out cases studies to compare typical lateral buckling mitigation techniques. Wang et al. [28] analysed pipeline laid on sleepers with numerical simulation method and gene expression programming technique. The simulation result show good accuracy comparing with the practice data.

These studies revealed the buckling characteristics of a pipeline with one imperfection. Because of the random distribution of initial imperfections, two or more initial imperfections may be located close together. In addition, there is little research on the buckling characteristics of a pipeline with multiple imperfections. This paper first calibrates the numerical simulation model from the axial force and lateral displacement perspectives, and then analyzes the buckling process of a pipeline with 2 imperfections by using this model. The load control criterion was used in this paper to judge whether the pipeline has failed, and the influence of 2 imperfections in the global buckling process was illustrated by comparing the failure determination parameters.

## The reliability verification of numerical simulation method

### The numerical simulation method for global buckling analysis

**Fig 1:**
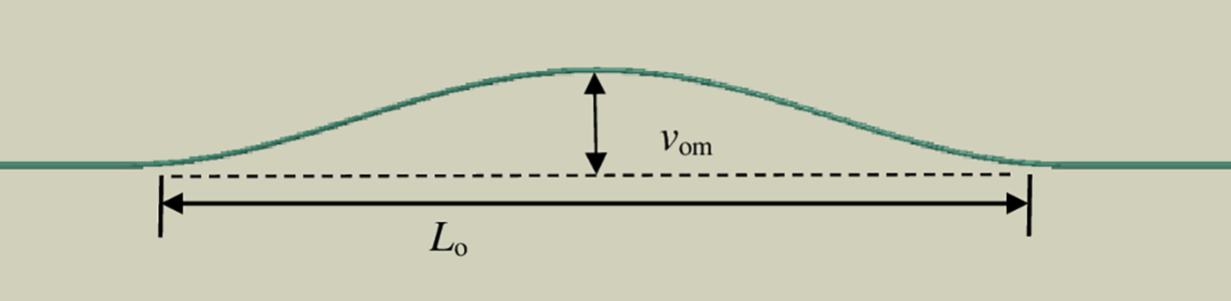
Buckle-Explicit united algorithm calculation model.

A global buckling simulation method called the Buckle-Explicit united algorithm is proposed for finite element analysis with the software ABAQUS. This method first calculates the most likely geometry feature of an initial imperfection based on the modal analysis results, and outputs the geometry of the imperfection through the “Nodefile” keyword. Then, this most likely imperfection is smoothly introduced into the buckling analysis with a certain size by editing the keyword of the global buckling simulation model. Fig 1 show a pipeline introduced with an initial imperfection by using Buckle-Explicit united algorithm.

The steel grade of the pipeline used in the finite element analysis is API 5L X65, and the outer diameter and thickness of the pipeline are 323.9 mm and 12.7 mm, respectively. The design temperature difference and pressure difference are 95°C and 4.65 MPa, respectively, and the thermal expansion coefficient of the pipeline steel is 1.1×10–5 /°C. The submerged weight of seabed soil is 7.8 kN/m^3^, and the cohesion of soil is 18 kPa. The internal friction angle of soil is 18.6°, and the pipe-soil friction coefficient is 0.4. The pipelines are simulated using PIPE31 elements. The pipeline is meshed using 0.5 m intervals in the axial direction. The initial imperfection is introduced in the middle of the pipeline. The seabed is 20 m wide, 1 m deep, and is simulated using C3D8R elements. The seabed is meshed using 0.5 m intervals in the axial direction, and the cross-section is divided into 20 meshes in the lateral direction and 2 meshes in the vertical direction. The sides of the seabed are constrained in the lateral and axial directions, and the bottom of the seabed is constrained in all three directions.

The pipe-soil interaction affects global buckling deformation heavily and many researchers did contribution to it. The soil resistance on partially embedded pipe [29–31], soil resistance during large pipeline movement [32] and influence of soil berm [33] are analyzed. The pipe-soil interaction in the Buckle-Explicit united algorithm is simulated as the friction between the pipeline and seabed soil, which is different from the spring connector used in earlier models. This pipe-soil interaction in the Buckle-Explicit united algorithm contains force in two directions: the normal direction and the tangential direction. The tangential restraint force is calculated based on the value of the normal contact force and the interaction coefficient. This interaction can also judge whether the pipeline and soil are connected or disconnected. The Buckle-Explicit united algorithm can adapt to the non-linear large deformation buckling simulation and analyze the structural dynamical response.

### The reliability verification of buckling simulation

The reliability verification of the buckling simulation method is carried out from two aspects: the result of lateral displacement and the result of axial force.

**Fig 2:**
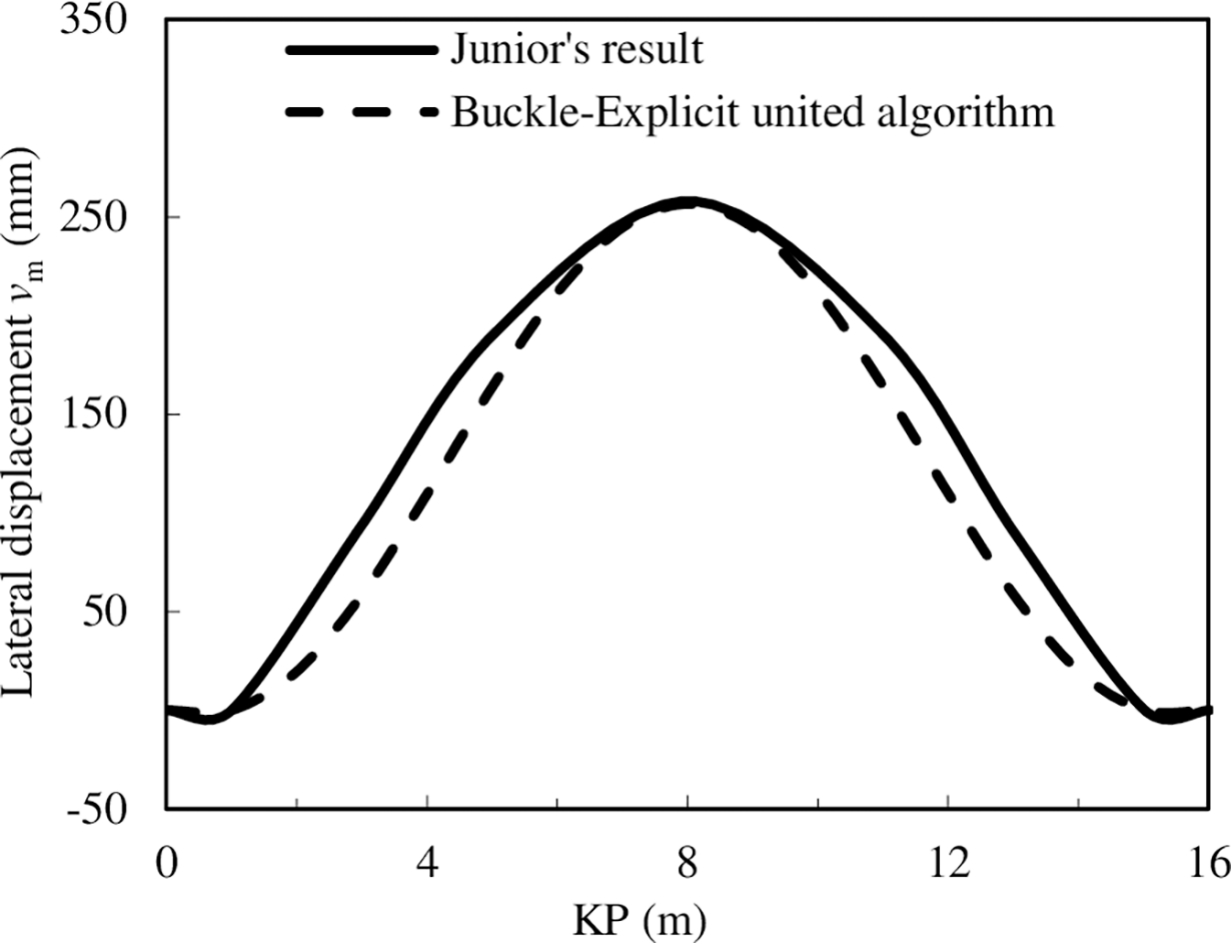
The comparison between the FEA result and the test result.

(1) The reliability verification from the lateral displacement: Junior et al. [34] carried out a large-scale pipeline global buckling test based on a pipeline failure case in Guanabara Bay. The test pipeline property is similar to the production pipeline, and the test records the deformation process of the test pipeline under temperature differences. This laboratory buckling test has the largest size so far and takes significant directive role in the real engineering. The test pipeline is 16 m in length, and the outer diameter and thickness are 76.2 mm and 2 mm, respectively. Thus, the Buckle-Explicit united algorithm is used to calculate the deformation of the test pipeline, and the simulation result is compared with the test result, as illustrated in Fig 2.

Fig 2 shows that the finite element analysis (FEA) result is similar to the test result. The biggest difference is located in the midpoint of the pipeline and the maximum displacement difference is less than 10%. Thus, the FEA result based on the Buckle-Explicit united algorithm is reliable.

**Fig 3:**
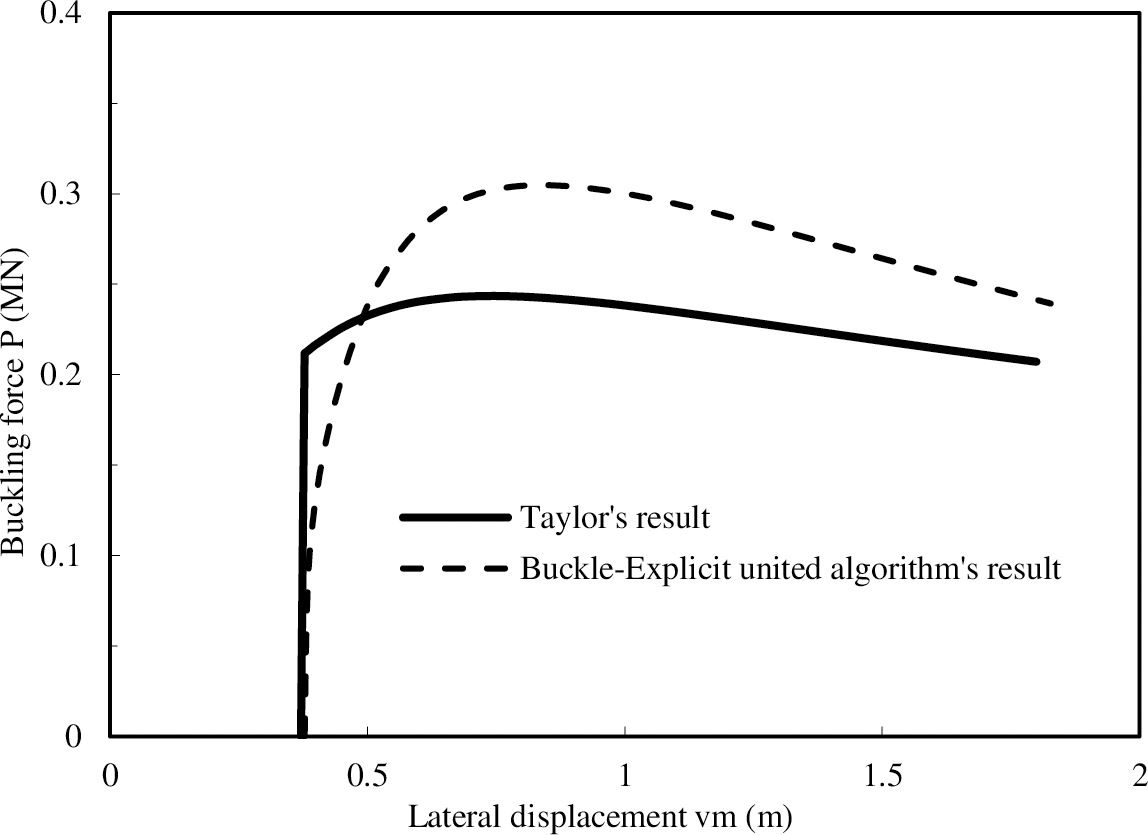
The comparison between the FEA result and the analytical result.

(2) The reliability verification from the axial force: Taylor and Gan [4] deduced the axial force analytical solution of pipeline buckling with one imperfection, and this solution is the classical solution in pipeline global buckling analysis. The Buckle-Explicit united algorithm is used to calculate the axial force of the same post-buckling pipeline analyzed in Taylor’s paper, and the result comparison between the two methods is displayed in Fig 3.

Fig 3 shows that the result from the Buckle-Explicit united algorithm is slightly higher than the analytical result, which is less than 20%. Considering that the computations of the two methods have little difference, we believe that the Buckle-Explicit united algorithm is accurate.

## The global buckling process of a pipeline with 2 initial imperfections

**Fig 4:**
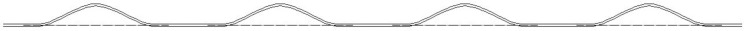
The shape of pipeline with multiple initial imperfections.

The Buckle-Explicit united algorithm is used for simulating the global buckling process of pipelines with 2 initial imperfections. One of the imperfection distributions is shown in Fig 4.

**Fig 5:**
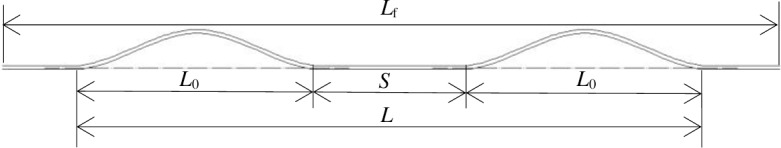
Feature parameters for a pipeline with two nearby imperfections.

Fig 4 illustrates that a pipeline with two proximate initial imperfections is the basic unit of a pipeline with multiple imperfections. Thus, a pipeline with two initial imperfections was chosen as the main object of research. The feature parameters for a pipeline with two nearby imperfections are displayed in Fig 5. The symbols *S* and *L*_0_ represent the initial imperfection’s space length and the imperfection’s wavelength, respectively; *L* represents the total length of the imperfection zone, which contains two imperfections and the spacing between them; and *L*_f_ represents the total length of the pipeline model.

**Fig 6:**
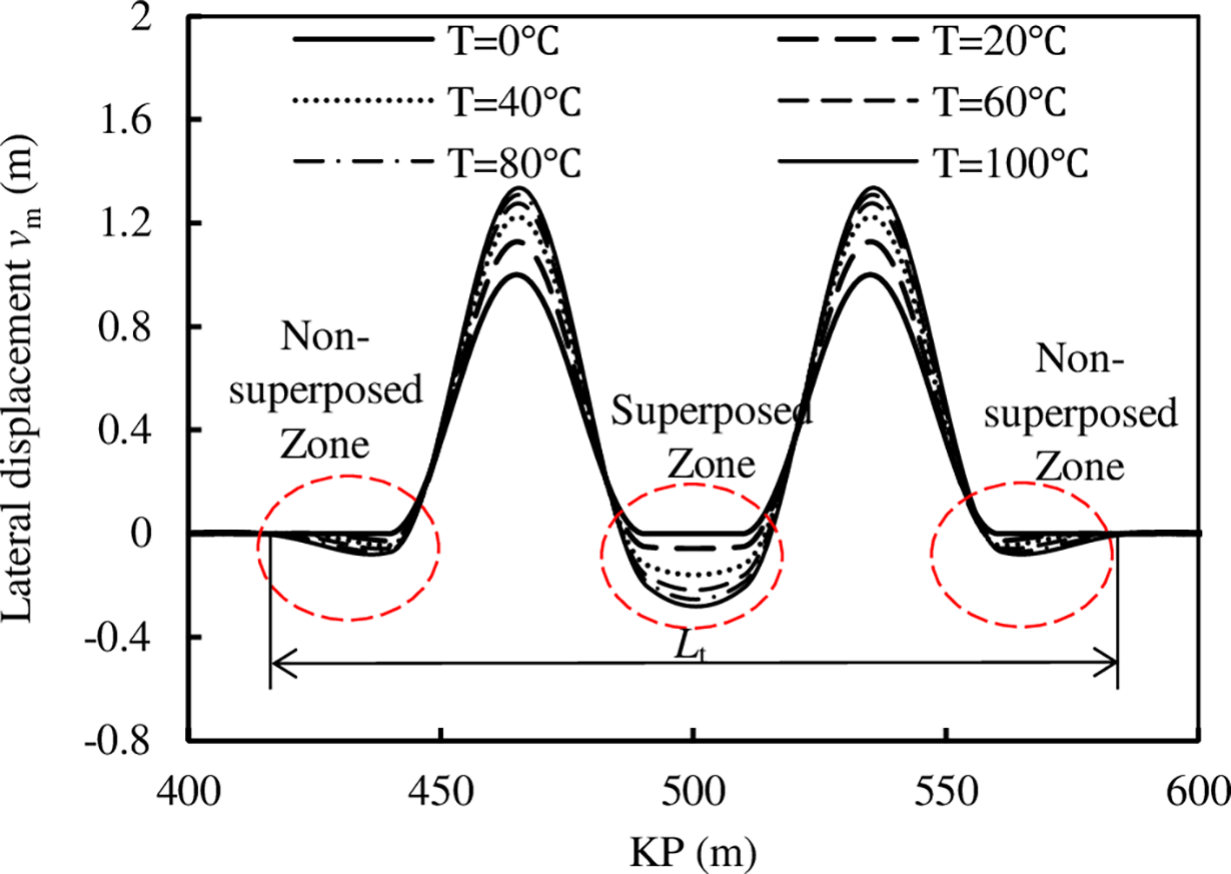
The lateral displacement variation in the lateral buckling process for pipeline with two imperfections.

To reveal the influence of 2 imperfections in the buckling process, a pipeline with 4.65 MPa pressure difference, and 100°C temperature difference was analyzed. The values of *S*, *L*_0_ and *L*_f_ are 20 m, 50 m and 1000 m, respectively. The axial force variation in the lateral buckling process is displayed in Fig 6 (the symbol *L*_t_ in Fig 6 indicates the length of the lateral deformed zone).

Fig 6 shows that the initial imperfections trigger the lateral buckling. The large lateral displacement occurred based on the initial imperfection, and the smaller lateral displacement in the opposite direction occurs at the ends of the initial imperfection. The lateral displacement is superposed between the two imperfections, and this part of the pipeline is called the superposition zone. The other parts of the pipeline that do not suffer displacement superposition are called the non-superposition zone. The displacement change rate with the temperature difference of the superposition zone is compared with the rate of the non-superposition zone. The result shows that the rate of the superposition zone is as 3.5 times larger than the rate of the non-superposition zone.

**Fig 7:**
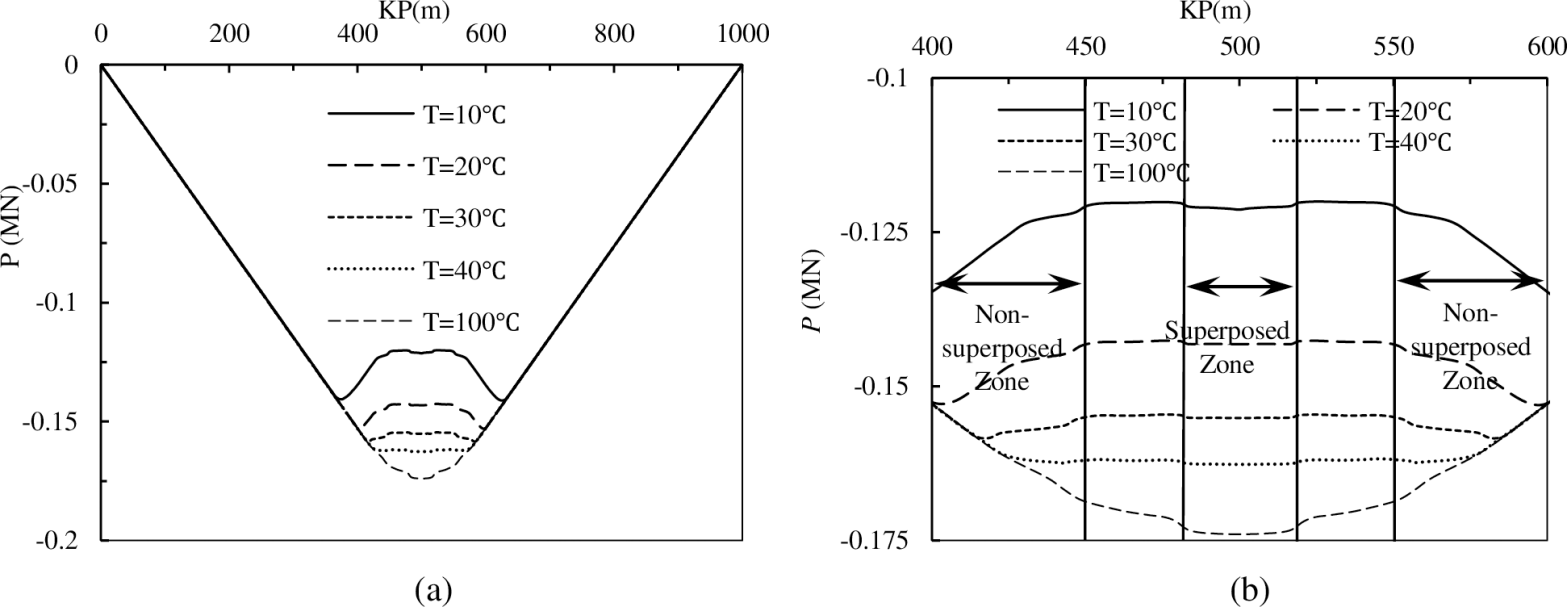
**The axial force variation in the lateral buckling process for pipeline with two imperfections**: (a) The axial force distribution along the whole pipeline; (b) The axial force distribution of the lateral deformed.

The variation of axial force in the lateral buckling process is displayed in Fig 7 (a negative value of axial force indicates that the force is a compressing force).

Fig 7(A) shows that when the temperature difference is 10°C, the axial force of the lateral deformed zone is much lower than the other parts. This is because the lateral deformation can release the accumulated axial compressive force. With the increasing temperature difference, the axial force of the lateral deformed zone increases and reaches a peak value in the midpoint of the pipeline. The results reveal that the initial imperfections slow the rate of accumulating axial force. Fig 7(B) displays that the axial force accumulating rate of the superposition zone is much faster than the non-superposition zone, and is approximately 1.5 times higher.

**Fig 8:**
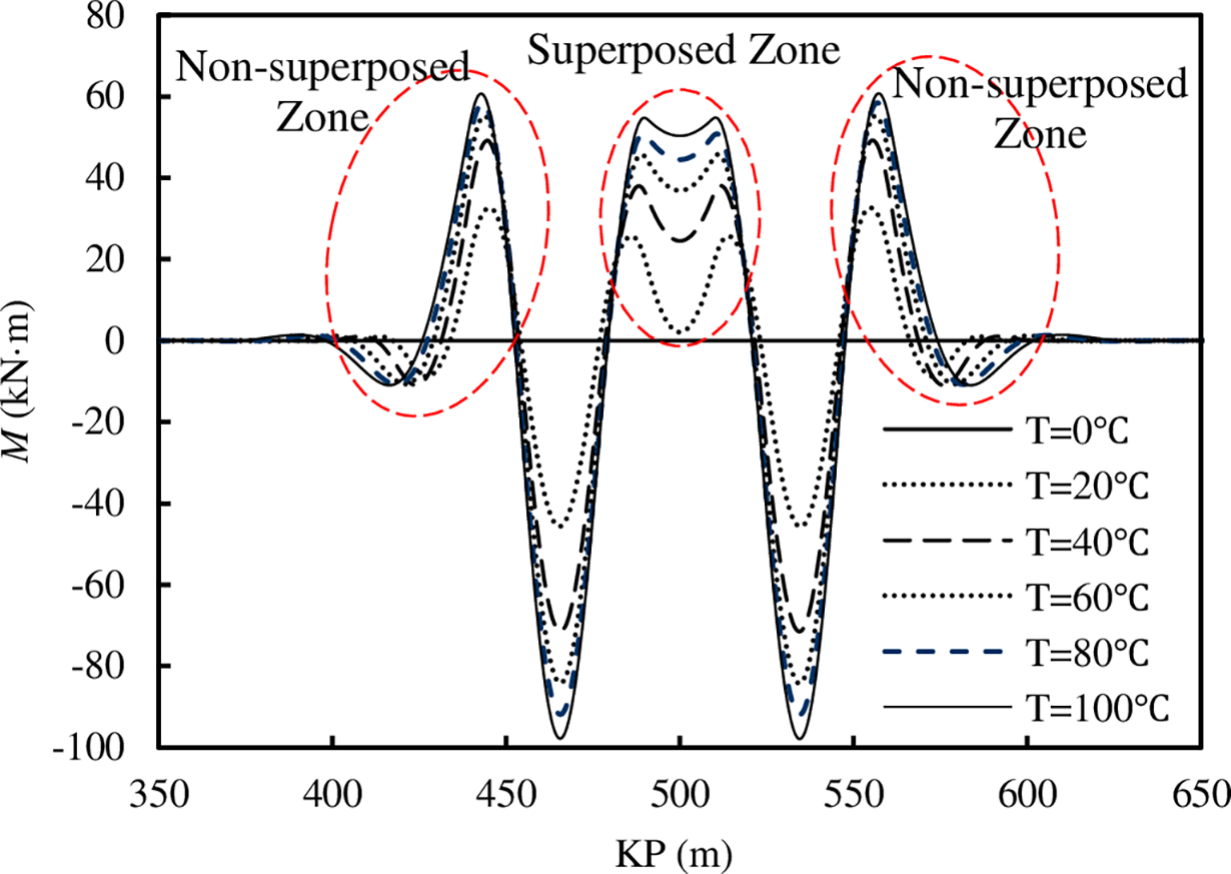
The bending moment variation in the lateral buckling process for pipeline with two imperfections.

The variation of the bending moment in the lateral buckling process is illustrated in Fig 8.

Fig 8 shows that the bending moment distribution along the whole pipeline is symmetric around the midpoint, and the bending moment increases with the rising temperature difference and increasing lateral deformation rate. The bending moment variation in the superposition zone is compared with the rate in the non-superposition zone, and the result illustrates that the bending moment change rate in the superposition zone is 7 times faster than the rate in the non-superposition zone.

The comparisons displayed above shows that the pipeline with 2 initial imperfections may suffer from the superposition of lateral global buckling, making the lateral displacement, axial force and bending moment increase much faster, and harming the stability of pipeline systems. Research on the lateral global buckling characteristics of pipelines with 2 imperfections is very necessary. For illustrating how the feature parameters of 2 imperfection combinations impact the buckling process, the sensitivity analysis of these parameters is carried out in the following section.

## The influence of 2 initial imperfections on pipeline lateral buckling

### Global buckling failure assessment coefficient

The global buckling failure of a post-buckling pipeline is assessed by estimating either the maximum internal force combination or the maximum compressive strain in the Det Norske Veritas code DNV-OS-F101. The assessment based on the maximum internal force combination is named the Load Control Condition, and the assessment based on the maximum compressive strain is named the Displacement Control Condition. The stricter Load Control Condition criterion is used in this paper to assess the global buckling failure of a post-buckling pipeline. The assessment judgement formula for the Load Control Condition is:

$${\left\{\gamma_{m}\gamma_{SC}\frac{M}{\alpha_{c}M_{p}}+{\left\{\frac{\gamma_{m}\gamma_{SC}P}{\alpha_{c}S_{p}}\right\}}^{2}\right\}}^{2}+{\left(\gamma_{m}\gamma_{SC}\frac{p_{e}}{p_{c}}\right)}^{2}\leq 1$$ (1)

where *γ*_m_ is the material coefficient of pipeline,0020*γ*_SC_ is the safety factor, *α*_c_ is the yield stress factor, *M* is the bending moment of the deformed pipeline cross-section, *M*_p_ is the equivalent yield bending moment, *P* is the axial force of deformed pipeline cross-section, *S*_p_ is the equivalent yield axial force, *α*_p_ is the section size coefficient, and *p*_b_ is the burst pressure. When (*p_i_*−*p_e_*)/*p_b_*≤2/3, *α*_p_ is *α_p_* = 1−*β*. Otherwise, *α*_p_ is *α_p_* = 1−3*β*⋅[1−*p_i_*−*p_e_*)/*p_b_*].

If the bending moment *M* and the axial force *P* of a post-buckling pipeline cross-section satisfies formula 1, then this pipeline is safe. Otherwise, the pipeline has experienced global buckling failure. The calculation value of the formula (1)’s left side can be defined as the global buckling failure assessment coefficient *θ*. The value range of *θ* is [0, 1], and the smaller the value of *θ*, the safer a pipeline is. The calculation formula of *θ* is:

$$\theta ={\left\{\gamma_{m}\gamma_{SC}\frac{M}{\alpha_{c}M_{p}}+{\left\{\frac{\gamma_{m}\gamma_{SC}P}{\alpha_{c}S_{p}}\right\}}^{2}\right\}}^{2}+{\left(\alpha_{p}\frac{p_{i}-p_{e}}{\alpha_{c}p_{b}(t)}\right)}^{2}$$ (2)

The meanings of the symbols in formula 2 are the same as the symbols in formula1. The variation of *θ* is calculated to estimate the influence of the initial imperfections’ space and the combination of the initial imperfections on the pipeline global buckling, and reveals the influence of the lateral buckling superposition on the stability of a pipeline.

### The influence of the initial imperfections’ space

To reveal the influence of the initial imperfections’ space on the superposition of global buckling, pipelines with different values of *S*/*L*_0_ are simulated. The symbol *S*/*L*_0_ represents the ratio of the initial imperfections’ space *S* to the imperfections’ length *L*_0_. A pipeline with 2 imperfections is displayed in Fig 5. The serial numbers of pipelines with different values of *S*/*L*_0_ are shown in Table 1.

**Table 1:** The serial numbers of pipelines with different values of *S*/*L*_0_.

NO.	S1	S2	S3	S4	S5	S6	S7
*S*/*L*_0_	0	0.5	0.8	1.0	1.2	1.4	2

**Fig 9:**
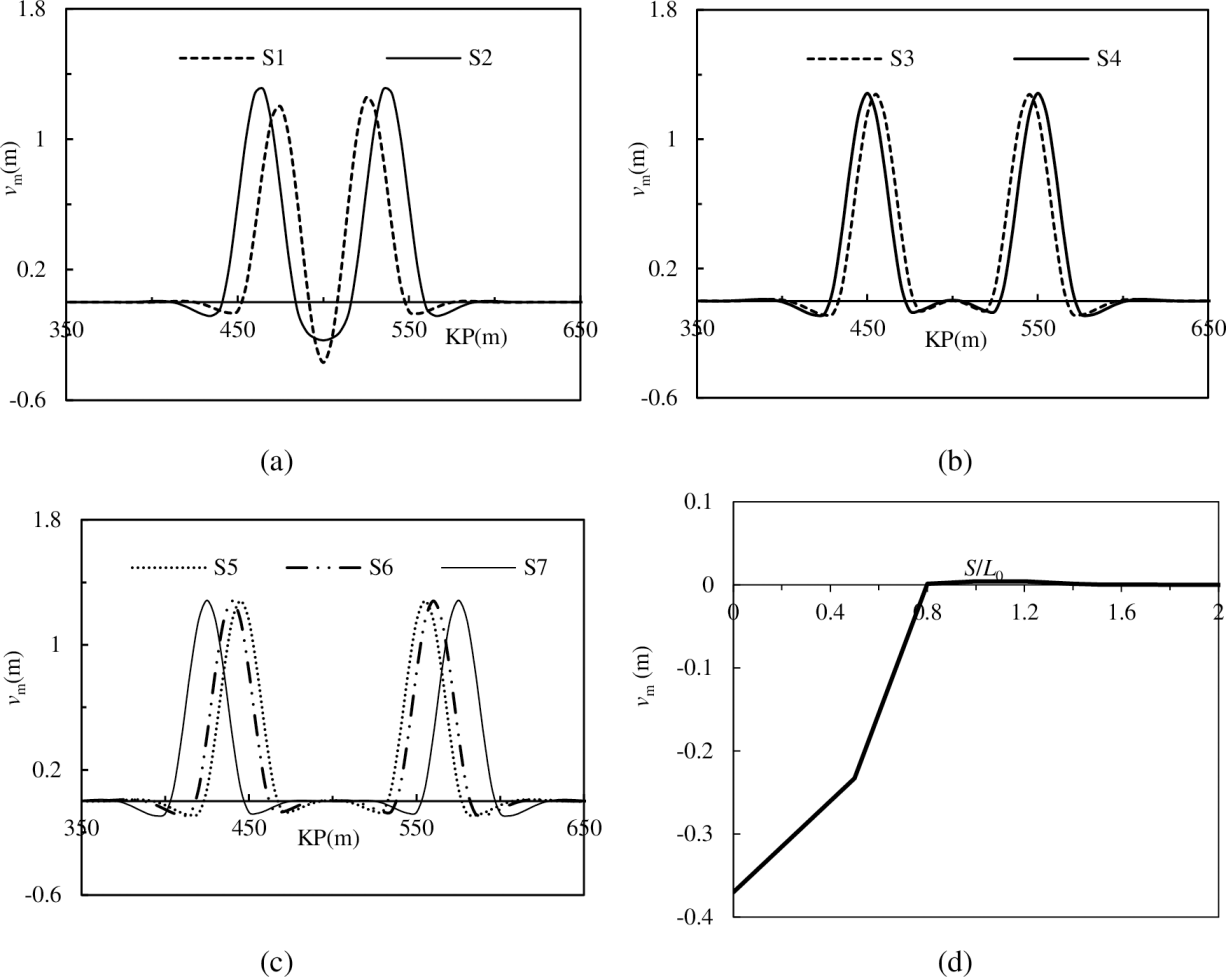
**The lateral deformation of pipelines with different spacing imperfections**: (a)The deformation of Model S1 and S2; (b) The deformation of Model S3 and S4; (c) The deformation of Model S5, S6 and S7; (d) Interaction between superposed lateral displacement and *S*/*L*_0_.

The models illustrated above are calculated, and these pipelines’ final lateral deformations are shown in Fig 9.

Fig 9(A), 9(B) and 9(C) show that the lateral displacement is trigged by the imperfections, and the superposition of global buckling occurs when the space of two imperfections is small enough (such as S1 and S2). Fig 9(D) shows that the lateral displacement of the superposed parts, which is located around the midpoint (KP = 500), decreases with increasing space. When the value of *S*/*L*_0_ is 0, the superposed lateral displacement reaches the peak value, and when the value of *S*/*L*_0_ increases to 0.8, the superposition of global buckling disappears. In other words, the critical value of *S*/*L*_0_ is 0.8 for this engineering condition.

**Fig 10:**
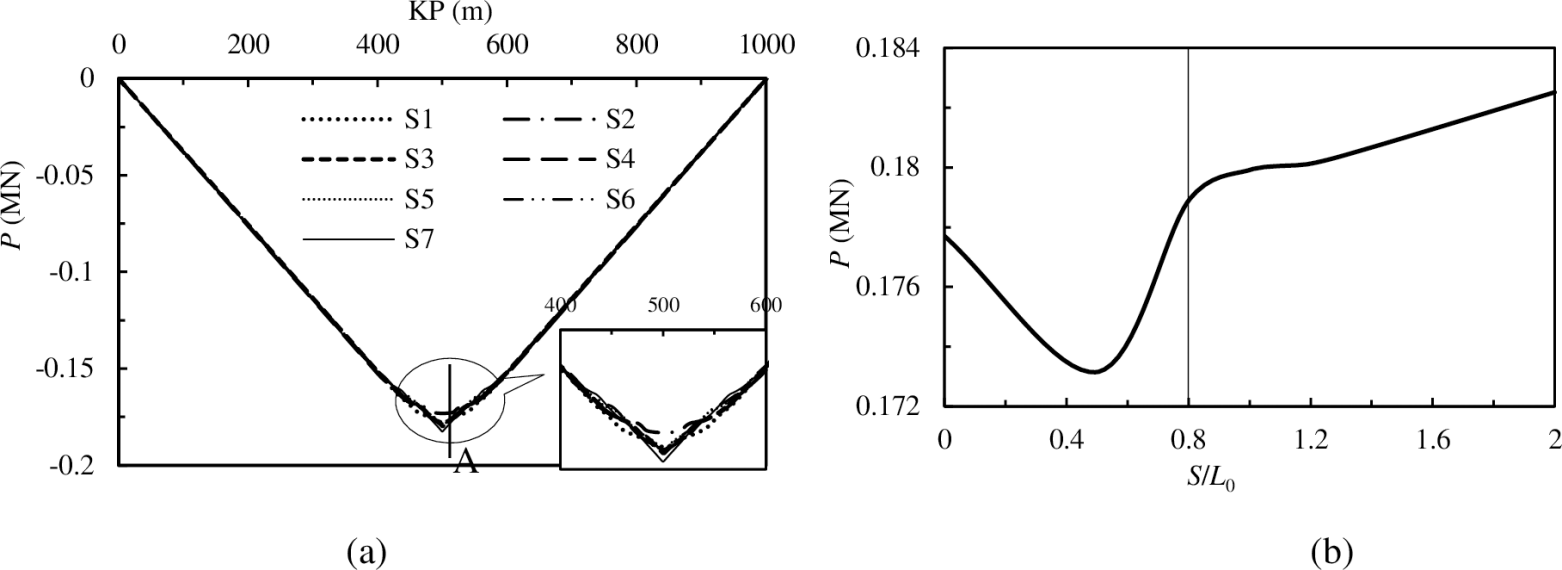
**The axial force of pipelines with different spacing imperfections**: (a) The variation of axial force along the pipeline; (b) Interaction between axial force of cross-section A and *S*/*L*_0_.

The distribution of pipelines’ axial forces for models S1-S7 and the axial force of cross-section A for these models are shown in Fig 10.

Fig 10(A) shows that the value of *S*/*L*_0_ slightly influences the axial force, and the location of the maximum axial force is in the middle of the pipeline for all 7 models. This cross-section is named cross-section A. Fig 10(B) illustrates that when *S*/*L*_0_ < 0.8, the axial force of cross-section A first decreases and then increases with increasing *S*/*L*_0_. When *S*/*L*_0_ > 0.8, axial force increases linearly with increasing *S*/*L*_0_, and the axial force of models with a *S*/*L*_0_ greater than 0.8 are all greater than the models with a *S*/*L*_0_ less than 0.8.

**Fig 11:**
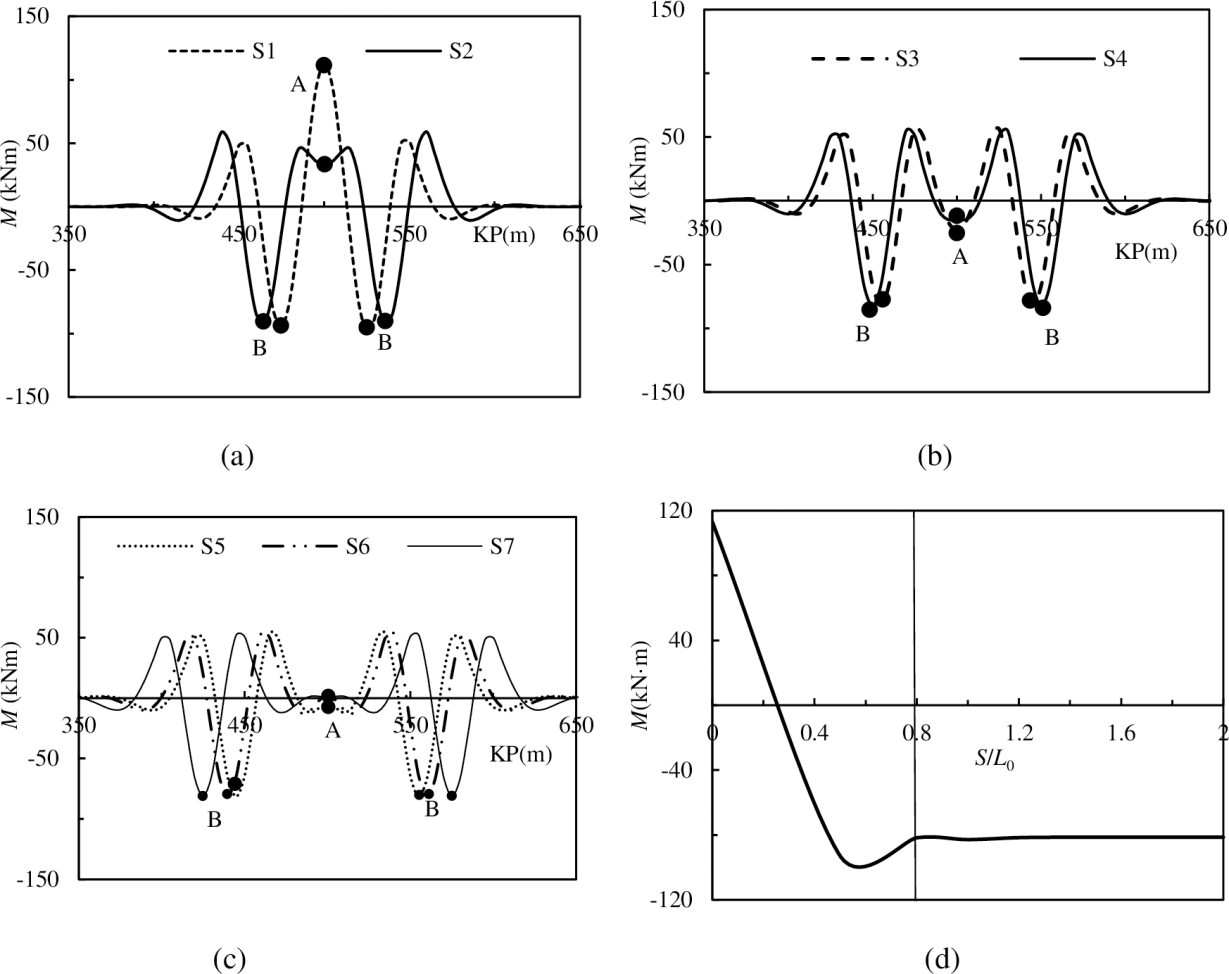
**The bending moment of pipelines with different spacing imperfections**: (a) The distribution of bending moment model S1, S2; (b) The distribution of bending moment for model S3, S4; (c) The distribution of bending moment for model S5, S6 and S7 (d) The maximum bending moment vs. *S*/*L*_0_.

The distribution of pipelines’ bending moments for models S1-S7 and the maximum bending moments for these models are shown in Fig 11.

Fig 11(A), 11(B) and 11(C) are the variations of the bending moments along the pipelines, and show that *S*/*L*_0_ influences the bending moments when pipelines suffer the superposition of global buckling. The superposed part suffers a greater bending moment, and the increment is basically proportional to the degree of lateral deformation superposition. When the value of *S*/*L*_0_ is 0, the bending moment reaches its peak value, and the cross-section suffering from the maximum bending moment is named cross-section B. The calculation results show that model S1 has one cross-section B. Models S2-S7 all have two cross-sections B, and these two cross-sections B are symmetric around the midpoint. Fig 11(D) is the interaction between the bending moment of cross-section B and *S*/*L*_0_, and it illustrates that *S*/*L*_0_ greatly influences the bending moment of cross-section B. When *S*/*L*_0_ < 0.8, the bending moment of cross-section B changes from a positive value to a non-linear negative value. Meanwhile, when *S*/*L*_0_ > 0.8, the bending moment of cross-section B changes little with increasing *S*/*L*_0_.

The global buckling failure assessment is based on the internal force combination; so, the axial force and bending moment for cross-section A and cross-section B are both used to calculate the failure coefficient *θ*. The larger one between *θ*_A_ represents the state of cross-section A and *θ*_B_ represents the state of cross-section B. which is chosen to assess the post-pipeline’s state. The calculation results are shown in Table 2.

**Table 2:** The global buckling failure coefficient *θ* for models S1-S7.

Model NO.	Cross-section	Internal force combination		Failure coefficient	Larger failure coefficient
		Axial force *P* (MN)	Bending moment *M* (kN·m)	*θ*_A_ (or *θ*_B_) (10^−3^)	*θ* (10^−3^)
S1	A(B)	-0.178	113.05	89	89
S2	A	-0.173	33.48	30	68
	B	-0.169	-92.84	68	
S3	A	-0.178	13.92	26	60
	B	-0.167	-81.74	60	
S4	A	-0.180	3.4	25	60
	B	-0.167	-82.85	60	
S5	A	-0.179	0.15	25	60
	B	-0.159	-81.65	60	
S6	A	-0.179	40.29	25	60
	B	-0.154	-81.40	60	
S7	A	-0.182	46.7	36	60
	B	-0.149	-81.40	60	

Table 2 illustrates that models S1-S7 are all safe in this condition and that the failure coefficient *θ* decreases with increasing *S*/*L*_0_. The pipeline with a larger *S*/*L*_0_ is safer. The comparison among the *θ* from models S1, S2 and S3 shows that the value of *S*/*L*_0_ changes from 0 to 0.5, the failure coefficient *θ* decreases 23.6%, and then the *S*/*L*_0_ continues to increase to 0.8, and *θ* again reduces to 11.8%. The comparison among the *θ* from models S4 to S7 shows that the failure coefficient *θ* does not change with increasing *S*/*L*_0_. A small value of *S*/*L*_0_ decreases the safety of a pipeline.

### The influence of combination

**Fig 12:**
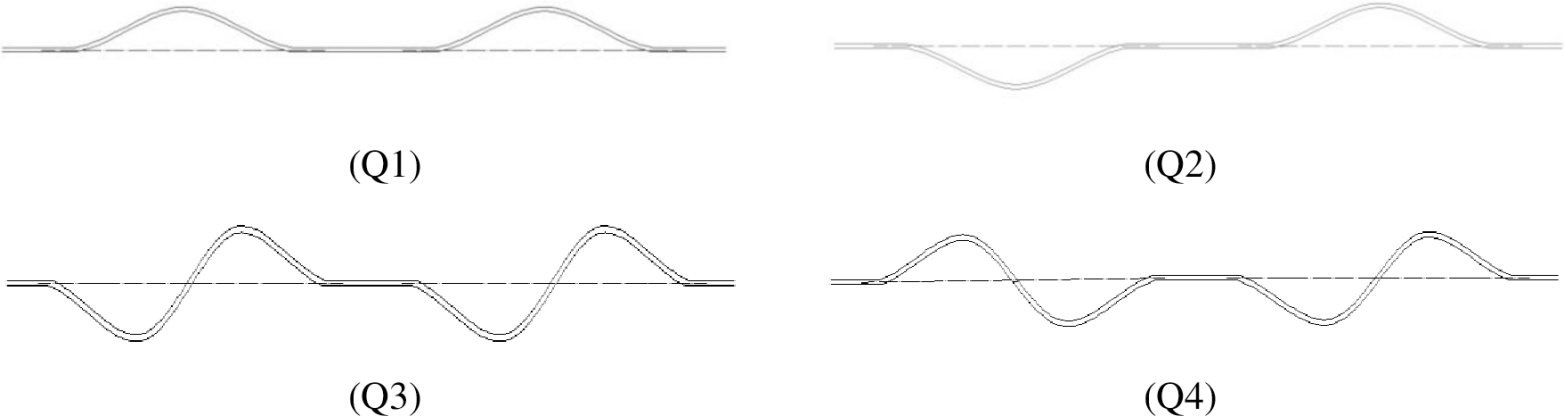
The combinations of imperfections used in the numerical analysis.

To improve the application of the analysis results, two kinds of typical imperfection forms are used in the combination influence analysis. The combinations of imperfections are illustrated in Fig 12.

The four combinations are numbered as follows: model Q1 is a pipeline with two single-arch imperfections in the same direction, and model Q2 is a pipeline with two single-arch imperfections in the opposite directions. Model Q1 and model Q2 have the same shape of imperfections. Model Q3 is a pipeline with two double-arch imperfections in the same direction, and model Q4 is a pipeline with two double-arch imperfections in the opposite directions. Model Q3 and model Q4 also have the same shape of imperfections.

**Fig 13:**
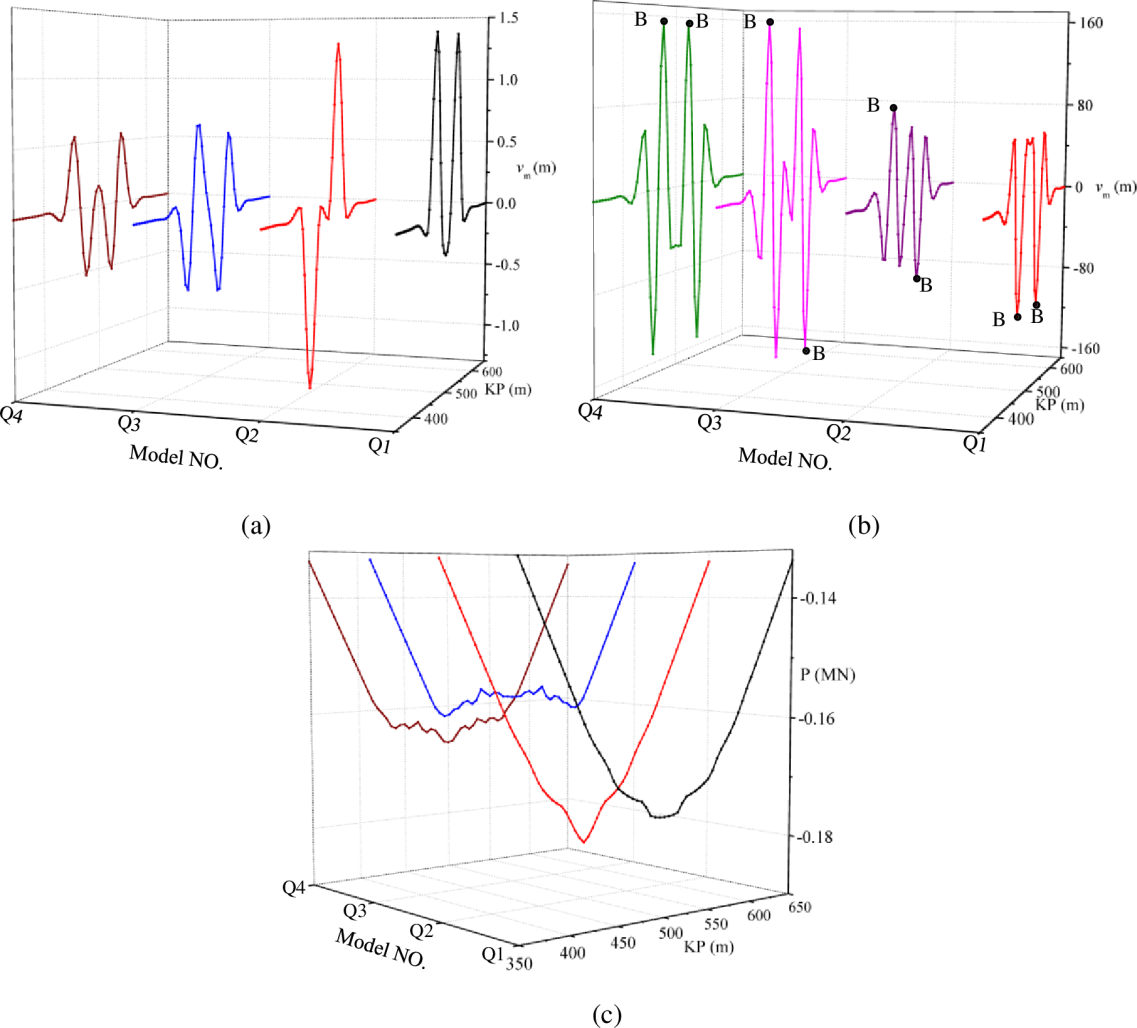
**Lateral global buckling characteristics for pipelines with different combination of imperfections**: (a) Lateral displacement; (b) Bending moment; (c) Axial force.

The numerical simulation models Q1-Q4 are calculated, and the lateral displacement, bending moment and axial force of each are displayed in Fig 13.

Fig 13(A) shows that the combination of imperfections has a major impact on lateral deformation. For pipelines with the same imperfection shapes, the pipelines with the same direction suffer larger lateral displacement than the pipelines with different directions, and the greatest difference is 21%. Meanwhile, the lateral displacement of the pipelines with double-arch imperfections is larger than the pipelines with single-arch imperfections in the same condition, and the greatest difference is 140%. For pipelines with single-arch imperfections, the superposition of lateral global buckling occurs when the imperfections are in the same direction. For pipelines with double-arch imperfections, the superposition of lateral global buckling occurs when the imperfections are in the opposite directions.

Fig 13(B) illustrates that for pipelines with the same imperfection shapes, the pipelines with the same direction suffer larger bending moments than the pipelines with different directions, and the greatest difference is 20%. The bending moments of the pipelines with double-arch imperfections are greater than the pipelines with single-arch imperfections in the same condition.

Fig 13(C) shows that for pipelines with the same imperfection shapes, the pipelines with same directions suffer less axial force than the pipelines with different directions, but the difference is small, being less than 4%. The axial forces of the pipelines with double-arch imperfections are less than the pipelines with single-arch imperfections in the same condition.

The axial forces and bending moments for cross-section A and cross-section B are both used to calculate the failure coefficient *θ*. The larger one between *θ*_A_ represents the state of cross-section A and *θ*_B_ represents the state of cross-section B, which is chosen to assess the post-pipeline’s state. The calculation results are shown in Table 3.

**Table 3:** The global buckling failure coefficient *θ* for models Q1-Q4.

Model NO.	Cross-section	Internal force combination		Failure coefficient	Larger failure coefficient
		Axial force *P* (kN)	Bending moment *M* (kN·m)	*θ*_A_ (or *θ*_B_) (10^−3^)	*θ* (10^−3^)
Q1	A	174	50.33	38	73
	B1	170	97.59	73	
Q2	A	180	0.005	25	58
	B1	173	81.53	58	
Q3	A	158	0.06	25	139
	B1	158	151.31	139	
Q4	A	167	56.02	41	145
	B1	164	154.90	145	

Table 3 illustrates that models S1-S7 are all safe in this condition and that the failure coefficient *θ* decreases with increasing *S*/*L*_0_. The pipelines with larger *S*/*L*_0_ are safer. The comparison among the *θ* from models S1, S2 and S3 shows that the value of *S*/*L*_0_ changes from 0 to 0.5, the failure coefficient *θ* decreases 23.6%, and then the *S*/*L*_0_ continues to increase to 0.8, and *θ* again reduces to 11.8%. The comparison among the *θ* from models S4 to S7 shows that the failure coefficient *θ* does not change with increasing *S*/*L*_0_. A small value of *S*/*L*_0_ decreases the safety of a pipeline.

Table 3 shows that models Q1-Q4 are all safe in this condition and that the failure coefficient *θ* of model Q2 is the smallest. The combination used in model Q2 is best for the safety of the pipeline among these four kinds of combinations. The comparison between model Q1 and model Q2 illustrates that the directions of two adjacent imperfections influence the value of *θ*, and the *θ* of a pipeline with same direction single-arch imperfections is 26% larger than a pipeline with opposite direction single-arch imperfections. The comparison between model Q3 and model Q4 illustrates that the *θ* of a pipeline with opposite directions imperfections is only 5% larger than a pipeline with same direction imperfections. Table 3 also illustrates that the *θ* of a pipeline with double-arch imperfections is larger than a pipeline with single-arch imperfections, and the maximum difference is 150%. The pipeline with opposite direction double-arch imperfections is most likely to experience global buckling failure.

## Conclusions

The ABAQUS/Explicit numerical simulation method is used to calculate the lateral deformation of pipelines with 2 imperfections. Based on the global buckling failure assessment recommended in the DNV code, the influences of imperfection space and combinations on pipeline global buckling failure are analyzed. The main conclusions are as follows:

1. A pipeline with two initial imperfections contains two global buckling deformed sections after pipeline is heated and pressured. If the space between the two imperfections is short enough, the two global buckling sections will interfere with each other. This buckling superposition makes lateral displacement, bending moment and axial force occur in the midpoint between the two imperfections.
2. Post-buckling pipeline failure state is also checked. The state of midpoint between two nearby imperfections (cross-section A) and the state of imperfection peak point (cross-section B) are both assessed. Results show pipeline exhibits global buckling superposition may failed at cross-section B, and the potential failure position is as same as no global buckling superposition pipeline. But the failure risk of pipeline exhibits global buckling superposition is several times greater than the failure risk of no global buckling superposition pipeline under the same operation condition.
3. The shape and direction of two nearby imperfections also affects the failure risk of pipeline exhibits global buckling superposition. Pipeline with two double-arch imperfections in opposite direction is most easy to fail after global buckling. And pipeline with two single-arch imperfections in opposite direction is least likely to fail after global buckling. The failure risk of pipeline with two double-arch imperfections is higher than pipeline with two single-arch imperfections.

## Supporting information

S1 File Detail data for Fig 2. (XLS)

S2 File Detail data for Fig 3. (XLS)

S3 File Detail data for Fig 6. (XLS)

S4 File Detail data for Fig 7. (XLS)

S5 File Detail data for Fig 8. (XLS)

S6 File Detail data for Fig 9. (XLS)

S7 File Detail data for Fig 10. (XLS)

S8 File Detail data for Fig 11. (XLS)

S9 File Detail data for Fig 13. (OPJ)

S10 File Language editing certificate. (PDF)

## References

[1] Sun J, Jukes P. From Installation to Operation: A Full-Scale Finite Element Modeling of Deep-Water Pipe-in-Pipe System. 28th International Conference on Ocean, Offshore and Arctic Engineering. American Society of Mechanical Engineers. 2009 439–446. doi: 10.1115/OMAE2009-79519

[2] Det Norske Veritas. Global Buckling of Submarine Pipelines—Structural Design Due to High Temperature/High Pressure. DNV-RP-F110. 2007.

[3] Det Norske Veritas. Submarine Pipeline Systems. DNV-OS-F101. 2007.

[4] Taylor N, Gan AB. Submarine pipeline buckling − imperfection studies. Thin-Walled Structures. 1986; 4(4): 295–323. doi: 10.1016/0263-8231(86)90035-2

[5] Taylor N, Tran V. Experimental and theoretical studies in subsea pipeline buckling. Marine Structures. 1996; 9(2): 211–257. doi: 10.1016/0951-8339(94)00021-J

[6] Croll JGA. A simplified model of upheaval thermal buckling of subsea pipelines. Thin-Walled Structures, 1997; 29(1): 59–78. doi: 10.1016/S0263-8231(97)00036-0

[7] Wantland GM, Oneill M, Reese LC, Kalajian EH. Lateral stability of pipelines in clay. Antimicrobial Agents & Chemotherapy. 1979; 45(3):936–7. doi: 10.4043/3477-MS

[8] Preston R, Drennan F, Cameron C. Controlled lateral buckling of large diameter pipeline by snaked lay. The 9th International Offshore and Polar Engineering Conference. 1999.

[9] Sriskandarajah T, Dong S, Sribalachandran S, Wilkins R. Effect of initial imperfections on the lateral buckling of subsea pipelines. The 9th International Offshore and Polar Engineering Conference. 1999; 2: 168–175.

[10] Villarraga JA, Rodrıguez JF, Martınez C. Buried pipe modeling with initial imperfections. Journal of pressure vessel technology. 2004; 126(2): 250–257. doi: 10.1115/1.1688369

[11] Bruton D, Carr M, Crawford M, Poiate E. The safe design of hot on-bottom pipelines with lateral buckling using the design guideline developed by the safebuck joint industry project. Proceedings of the Deep Offshore Technology Conference, Vitoria, Espirito Santo, Brazil. 2005.

[12] Sparks CP. The influence of tension, pressure and weight on pipe and riser deformations and stresses. Journal of Energy Resources Technology. 1984; 106(1): 46–54. doi: 10.1115/1.3231023

[13] Fyrileiv O, Collberg L. Influence of pressure in pipeline design: effective axial force. 24th International Conference on Offshore Mechanics and Arctic Engineering. American Society of Mechanical Engineers. 2005; 629–636. doi: 10.1115/OMAE2005-67502

[14] Bruton DAS, Carr M, White DJ. The influence of Pipe-Soil interaction on Lateral Buckling and walking of pipelines- The SAFEBUCK JIP. Proc. International Offshore Investigation and Geotechnics (OSIG) Conference. 2007.

[15] Bruton D, Bolton M, Carr M, White DJ. Pipe-soil interaction with flowlines during lateral buckling and pipeline walking—The SAFEBUCK JIP. Offshore Technology Conference. 2008. doi: 10.4043/19589-MS

[16] Sun J, Jukes P, Wang J. The advancements of FEA in confronting the deepwater pipelines under high pressure and high temperature. Offshore Technology Conference. 2011. doi: 10.4043/22306-MS

[17] Chee K Y and Walker A. Assessment of Numerical Modelling of Pipeline Lateral Buckling Proceeding of International Symp. on Lateral Buckling, Australia 2011.

[18] Konuk I, Yu S. Continuum FE modeling of lateral buckling: study of soil effects. 26th International Conference on Offshore Mechanics and Arctic Engineering. 2007; 347–354. doi: 10.1115/OMAE2007-29376.

[19] Newson TA, Deljoui P. Finite element modelling of upheaval buckling of buried offshore pipelines in clayey soils. GeoShanghai International Conference 2006. doi: 10.1061/40862(194)47

[20] Haq MM, Kenny S. Lateral buckling response of subsea HTHP pipelines using finite element methods. 32nd International Conference on Ocean, Offshore and Arctic Engineering. 2013. doi: 10.1115/OMAE2013-10585

[21] Liu R, Liu WB, Wu X, Yan SW. Global lateral buckling analysis of idealized subsea pipelines. Journal of Central South University. 2014; 21: 416–427. doi: 10.1007/s11771-014-1955-y

[22] Hong ZH, Liu R, Liu WB, Yan SW. A lateral global buckling failure envelope for a high temperature and high pressure (HT/HP) submarine pipeline. Applied Ocean Research, 2015; 51:117–128. doi: 10.1016/j.apor.2015.02.008

[23] Hong ZH, Liu R, Liu WB, Yan SW. Study on lateral buckling characteristics of a submarine pipeline with a single arch symmetric initial imperfection. Ocean engineering. 2016; 108: 21–32. doi: 10.1016/j.oceaneng.2015.07.049

[24] Li G, Zhan LC, Li H. An analytical solution to lateral buckling control of subsea pipelines by distributed buoyancy sections. Thin-Walled Structures, 2016; 107: 221–230. doi: 10.1016/j.tws.2016.06.003

[25] Zhang M, Yu JX, Sun ZZ, Fang ZY, Wu MN, Duan JH. Application research of Patran and Abaqus in offshore pipeline buckling analysis. The Chinese journal of Ocean Engineering, 2016; 34(3): 55–62. doi: 10.16483/j.issn.1005-9865.2016.03.007

[26] Sriskandarajah T, Roberts G, Tanscheit P, Solano RF, Hansen A, Antunes BR. Forward Predictions for Life-of-Field Assessments Based on the Observed Lateral Buckling Behavior of Operating Deepwater Pipelines. Offshore Technology Conference 2015. doi: 10.4043/25961-MS

[27] Hakim MA, Azouz A, Awda H. To Buckle or Not to Buckle—Best Practices for HP/HT Pipelines Lateral Buckling Design in Shallow Water. Offshore Technology Conference 2016. doi: 10.4043/27095-MS

[28] Wang Z, Chen ZH, Liu HB, Zhang ZC. Numerical study on lateral buckling of pipelines with imperfection and sleeper. Applied Ocean Research, 2017; 68:103–113. doi: 10.1016/j.apor.2017.08.010

[29] Lee YS, Cheuk CY, Smith CC. Lateral Soil Resistance for On-bottom Pipeline Design on Clayey Seabed. HKIE Transactions, 2013; 19(4):2–10. doi: 10.1080/1023697X.2012.10668999

[30] Figueiredo FC, Borges LA, Pontes IS, Costa L. Limit analysis on evaluation of lateral resistance of partially embedded pipes with frictional pipe-soil interface. 8th International Conference on Computational Plasticity. Fundamentals and Applications. 2015.

[31] Mccarron W. Limit Analysis and Finite Element Evaluation of Lateral Pipe-Soil interaction resistance. Canadian Geotechnical Journal, 2015; 53(1): 150610143422006. doi: 10.1139/cgj-2014-0409

[32] Randolph M, White DJ, Chatterjee S. Numerical simulations of pipe–soil interaction during large lateral movements on clay. Géotechnique, 2012; 62(8):693–705. doi: 10.1680/geot.10.P.107

[33] Wang L, Liu R. The effect of a berm on the lateral resistance of a shallow pipeline buried in sand. Ocean Engineering, 2016; 121:13–23. doi: 10.1016/j.oceaneng.2016.05.010

[34] Junior EP, Rocha RSD, Álvaro MDC, Guimaraes GB, Amral C, Souza PFD. Experimental Tests and Numerical Simulation in a Reduced Model in a Pipeline With ZIG-ZAG Geometry: A Parametric Study. International Pipeline Conference. 2004. 399–407. doi: 10.1115/IPC2004-0423

